# Reconstruction giant cell tumor of the right proximal humerus Campanacci 3 with pedicle and rod system: A case report

**DOI:** 10.1016/j.ijscr.2018.08.042

**Published:** 2018-08-29

**Authors:** Yogi Prabowo, Irsan Abubakar

**Affiliations:** a Musculoskeletal Oncology Division, Department of Orthopaedic & Traumatology, Cipto Mangunkusumo National Central Hospital and Faculty of Medicine, Universitas Indonesia, Jalan Diponegoro No. 71, Central Jakarta, Jakarta 10430, Indonesia; b Department of Orthopaedic & Traumatology, Cipto Mangunkusumo National Central Hospital and Faculty of Medicine, Universitas Indonesia, Jalan Diponegoro No. 71, Central Jakarta, Jakarta 10430, Indonesia

**Keywords:** Giant cell, Campanacci 3, Wide excision, Reconstruction, Pedicle screw, Rod system

## Abstract

**Introduction:**

Giant Cell tumors (GCT) are benign tumors with potential for aggressive behavior and capacity to metastasize. Although considered to be benign tumors of bone, GCT has a relatively high recurrence rate. Tumor often extends to the articular subchondral bone or even abuts the cartilage. The joint and/or its capsule are rarely invaded. Surgical resection is the universal standard of care for the treatment of bone GCT. The key ensuring an adequate surgical treatment with complete removal of tumor is by obtaining adequate exposure of the lesion.

**Presentation of case:**

We reported a case of 24-years-old male with Giant cell tumor (GCT) of the right proximal humerus. Patient presented with chief complaint of pain on the right shoulder and had a history of fell on the right elbow. Radiographic examination showed a primary bone tumor of the proximal humerus. MRI provided excellent depiction in suggesting the diagnosis of cutaneous GCT Campanacci 3, which was later, affirmed by biopsy. Patient underwent successful wide excision and reconstruction. The limb salvage procedure consisted of shoulder resection type 1B and reconstruction with pedicle screw and rod system. During 5 days post-operative period, there was no major event observed. Patient could do shoulder flexion forward 0–30, shoulder extension 0–20, elbow extension – Flexion, wrist flexion extension, and fingers flexion.

**Discussion:**

GCT of bone typically shown as an epiphyseal, eccentric, expansive lytic lesion with a ‘soap-bubble appearance’. MRI is useful to assess extracortical spread and intramedullary extension. Surgery is the treatment of choice. Curettage is usually combined with cementing or bone grafting. Hemi-articular and total elbow allografts have been used for reconstruction of the defects following tumor excision, but the complication rates are high, and these techniques are reserved as salvage procedures following failed total elbow arthroplasty.

**Conclusion:**

Wide resection and total elbow arthroplasty enables good functional outcome and lower risk for recurrence. Pedicle and rod system for shoulder reconstruction is a viable option, as it provides good pain relief and functional improvement with lower complication rates.

## Highlights

• Giant Cell tumors (GCT) are benign tumors with potential for aggressive behavior and capacity to metastasize.
• GCT were classified by Enneking and later by Campanacci based on radiographic appearance.
• Wide resection is associated with decreased risk of local recurrence compared to intralesional curettage and may increase the recurrence free survival rate.
• A 24-years-old male presented with Giant cell tumor (GCT) of the right proximal humerus Campanacci 3 and underwent wide resection and reconstruction type 1B with pedicle screw and rod system.
• The procedure provided excellent local control as the outcome was good both aesthetically and functionally.

## 1 Introduction

**Fig. 1:**
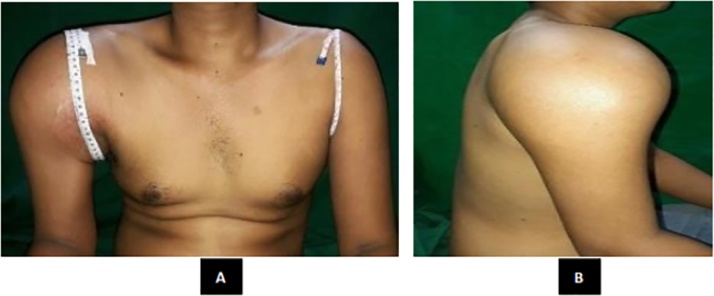
Physical examination of the right shoulder (**A**) Anterior view. (**B**) Lateral view.

Giant Cell tumors (GCT) are benign tumors with potential for aggressive behavior and capacity to metastasize. GCT represents approximately 5% of all primary bone tumors [1,2]. Although rarely lethal, benign bone tumors may be associated with a substantial disturbance of the local bony architecture that can be particularly troublesome in peri-articular locations [2]. Although considered to be benign tumors of bone, GCT has a relatively high recurrence rate. Metastases occur in 1%–9% of patients with GCT and some earlier studies have correlated the incidence of metastases with aggressive growth and local recurrence [3]. The prevalence of GCT peaks during the 3rd decade, with 80% of cases occurring between 20 and 50 years of age. Less than 3% of cases occur before the age of 14 years, and only 13% of cases occur in patients over the age of 50 years [4] (Fig. 1).

**Fig. 2:**
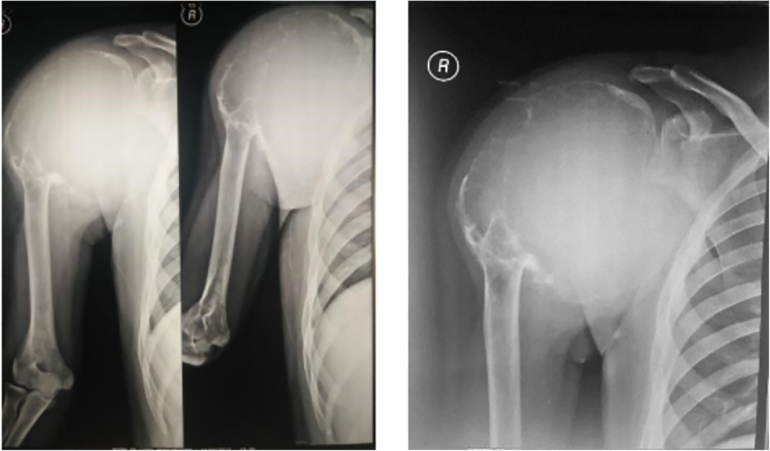
Anteroposterior and Lateral radiograph of shoulder depicts soft tissue tumor.

Ninety percent of GCT exhibits a typical epiphyseal location. Tumor often extends to the articular subchondral bone or even abuts the cartilage. The joint and/ or its capsule are rarely invaded. In rare instances in which GCT occurs in a skeletally immature patient the lesion is likely to be found in the metaphysis [5]. The most common locations, in decreasing order, are the distal femur, the proximal tibia, the distal radius, and the sacrum. Fifty percent of GCTs arise around the knee region. Other frequent sites including the fibular head, the proximal femur, and the proximal humerus. Pelvic GCT is rare [6] (Fig. 2).

**Fig. 3:**
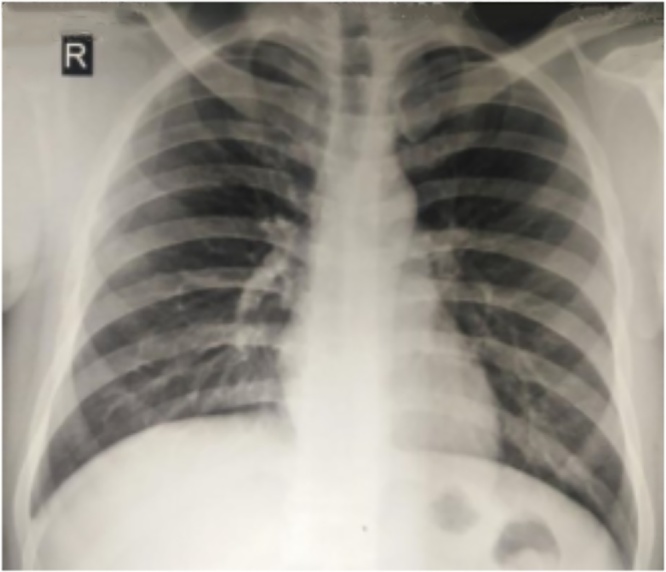
Chest radiograph showed no lung metastases.

Pain is the leading symptom related to the mechanical insufficiency resulting from the bone destruction. A soft tissue mass or bump can occasionally be seen and resulted from the cortical destruction and tumor progression outside the bone. GCT is often found close to the joint thus limiting range of motion. Joint effusion and synovitis are also possible. At diagnosis, approximately 12% of patients with GCT present with pathologic fracture [7]. Presentation with a pathologic fracture is thought to indicate a more aggressive disease with a higher risk of local recurrence and metastatic spread [7,8] (Fig. 3).

**Fig. 4:**
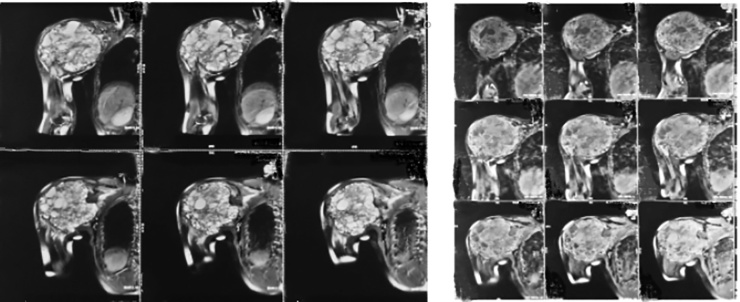
Magnetic Resonance Imaging showing an expansile lytic lesion.

GCT were classified by Enneking and later by Campanacci based on radiographic appearance. They described three stages that correlate with tumor local aggressiveness and risk of local recurrence, Stage I (latent), Stage II (active), Stage III (aggressive). Campanacci attempted to grade the lesions based on radiological appearance. All of the tumors, both primary and recurrent, are graded radiographically, using the designations Grade I, Grade II, Grade II with fracture, and Grade III. Grade I tumor has a well-margined border of a thin rim of mature bone, and the cortex is intact or slightly thinned but not deformed. Grade II tumor has relatively well-defined margins but no radiopaque rim; the combined cortex and rim of reactive bone is rather thin and moderately expanded but still present. Grade II lesions with a fracture are graded separately. Grade III designates a tumor with fuzzy borders, suggesting a rapid and possible permeative growth; the tumor bulges into the soft tissues, but the soft tissue mass does not follow the contour of the bone and is not limited by an apparent shell of reactive bone (Fig. 4).

**Fig. 5:**
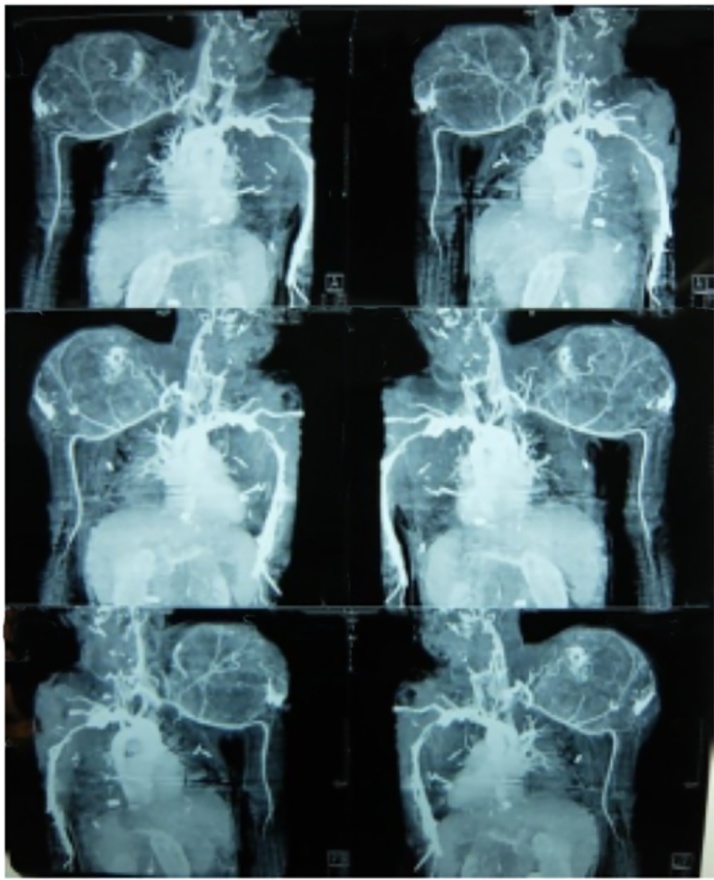
CT Angiography.

Surgical resection is the universal standard of care for treatment of GCT of bone. As most giant cell tumors are benign and are located near a joint in young adults, several authors favor an intralesional approach that preserves anatomy of bone in lieu of resection [9,10]. Various studies suggested that wide resection is associated with decreased risk of local recurrence compared to intralesional curettage and may increase the recurrence free survival rate from 84% to 100% [11]. However, wide resection is associated with higher rates of surgical complications and leads to functional impairment, generally necessitating reconstruction [11,12]. Local control without sacrificing joint function has traditionally been achieved by intralesional curettage with autograft reconstruction by packing the cavity of the excised tumor with morsellised iliac cortico cancellous bone. Regardless of how thoroughly performed, intralesional excision leaves microscopic disease and hence has a reported recurrence rate as high as 60% (Fig. 5).

**Fig. 6:**
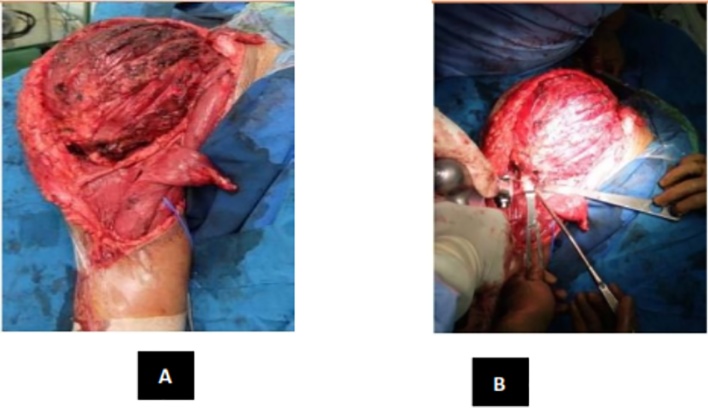
(**A**) Tumor Exposed (**B**) Distal Humerus Osteotomy.

The key ensuring an adequate curettage with complete removal of tumor is by obtaining adequate exposure of the lesion [13]. This is best achieved by making a large cortical window to access the tumor so as to avoid having to curette under overhanging shelves or ridges of bone. Use of a headlamp and dental mirror combined with multiple angled curettes help to identify and access small pockets or residual disease, which may otherwise result in recurrence. A high-power burr to break the bony ridges helps extend the curettage. A pulsatile jet-lavage system used at the end of the curettage helps to bare raw cancellous none and physically washout tumor cells [14]. Historically, the rate of local recurrence after curettage alone and bone grafting has been reported to range between 25% and 50% [15]. This has led surgeons to enhance their surgical procedure with use of chemical or physical adjuvants such as liquid nitrogen, acrylic cement, phenol, hydrogen peroxide, locally delivered chemotherapy, or radiation therapy [16,17]. The latter has been linked with malignant transformation in the past but the risk of this complication has been recently challenged and may be different with modern radiotherapy modalities [18]. Local adjuvant therapy has been shown to be useful in controlling recurrence rates (Fig. 6). The literature has shown 6%–25% recurrence rates in GCT treated with curettage and local adjuvant therapy [19]. Having described that, recent studies have questioned the role of adjuvants and filling agents in reducing the recurrence rate of GCT, they infer that adequate removal of the tumor seems to be a more important predictive factor for the outcome of surgery than the use of adjuvants.

Trieb demonstrated that local recurrence rate of GCT located in long bones treated with or without phenol is similar [15]. Prosser recommended primary curettage for intra-osseous GCT without adjuvant treatment or filling agents [20]. Reconstructing the defect after curettage can be quite a challenge. If the gap left behind after the curettage is small and does not jeopardize the structural integrity of bone it can be left alone and the cavities fill up with blood clot, which then gets ossified to form bone. For larger defects the traditional methods of reconstruction have been cementation or use of bone graft.

This work has been reported in line with the SCARE criteria [21].

## 2 Presentation of case

**Fig. 7:**
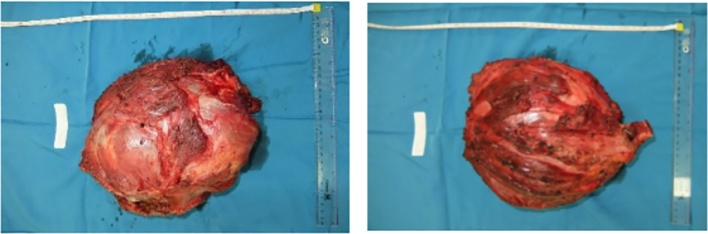
Tumor Mass.

A 24-years-old Male presented with chief complaint pain on the right shoulder since 11 month ago. Patient had a past history of falling on the right elbow hit the ground first. The patient directly felt pain from the elbow radiated to the shoulder. The patient couldn’t move the shoulder and then went to masseuse for the problem. A month later the shoulder became swollen and grew to the size of a ball. Five months ago, the patient went to General Hospital and had an X-ray examination resulted in a bone tumor. The patient was referred to another General Hospital and then to our hospital (Fig. 7).

**Fig. 8:**
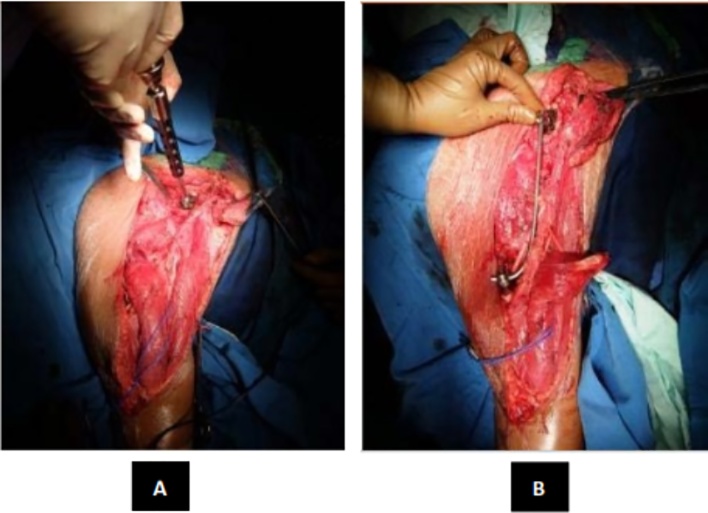
(A) Pedicle Screw Insertion (B) Rod Application.

On local examination, there was a lump on the right shoulder. The tumor had a hard consistency with well-defined margin, no sinus, no ulcer, and no venectation. Skin was intact. No distal oedema was observed. The local temperature was slightly increased and the mass was tender (visual analogue scale of 2–3). The capillary refill time was under 2 s, distal pulsation was still palpable and sensation was normal. There was restriction of range of movement of the shoulder (Fig. 8).

During further assessment, plain radiography was performed, showing soft tissue tumor on the right shoulder. Radiographs showed an expansive lytic lesion with ‘soap bubble’ appearance. We also performed a chest radiograph to rule out pulmonary metastasis.

Magnetic Resonance Imaging was requested in this case to determine the entity of this tumor. CT Angiography was used to know the involvement of the axillary artery.

The patient had undergone core biopsy, and investigation was reviewed in Pathology of Anatomy, of our institution. According to the investigation, the result was Giant Cell Tumor with Aneurysmal bone cyst. This case was brought and discussed in the Clinicopathological Conference (CPC – A board consisting of experts from Orthopaedics, Radiology, and Pathology Anatomy Department – and the patient was decided for wide excision and reconstruction with pedicle screw and rod system.

**Fig. 9:**
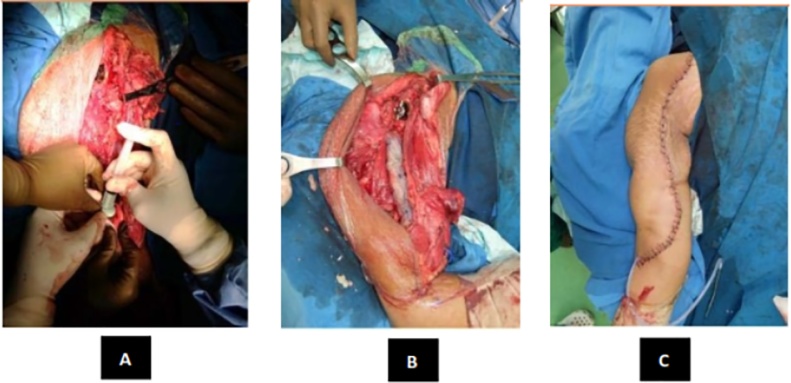
(A) Bone Cement Application (B) Mess Application (C) Wound Closure. Post-operative radiograph was taken and showed (Fig. 10).

Intraoperative, we found the tumor was easily demarcated. The glenohumeral joint of this patient was destroyed. After the tumor was exposed, we evaluated the margin of the wound, then we done distal humerus osteotomy and glenohumeral disarticulation. Margin of tumor excision that we done was tumor free. After the tumor was removed, we inserted Axial pedicle screw in glenoid, pedicle mono axial in intramedullary distal humerus and rod application. Finally, we decided to perform primary wound closure (Fig. 9).

**Fig. 10:**
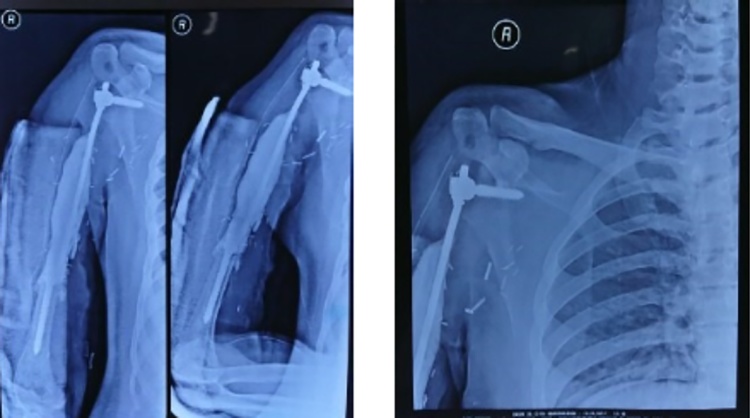
Postoperative radiograph. Post-operative period was good, patient can do elbow extension and wrist flexion extension. Post-operative clinical view (Fig. 11).

**Fig. 11:**
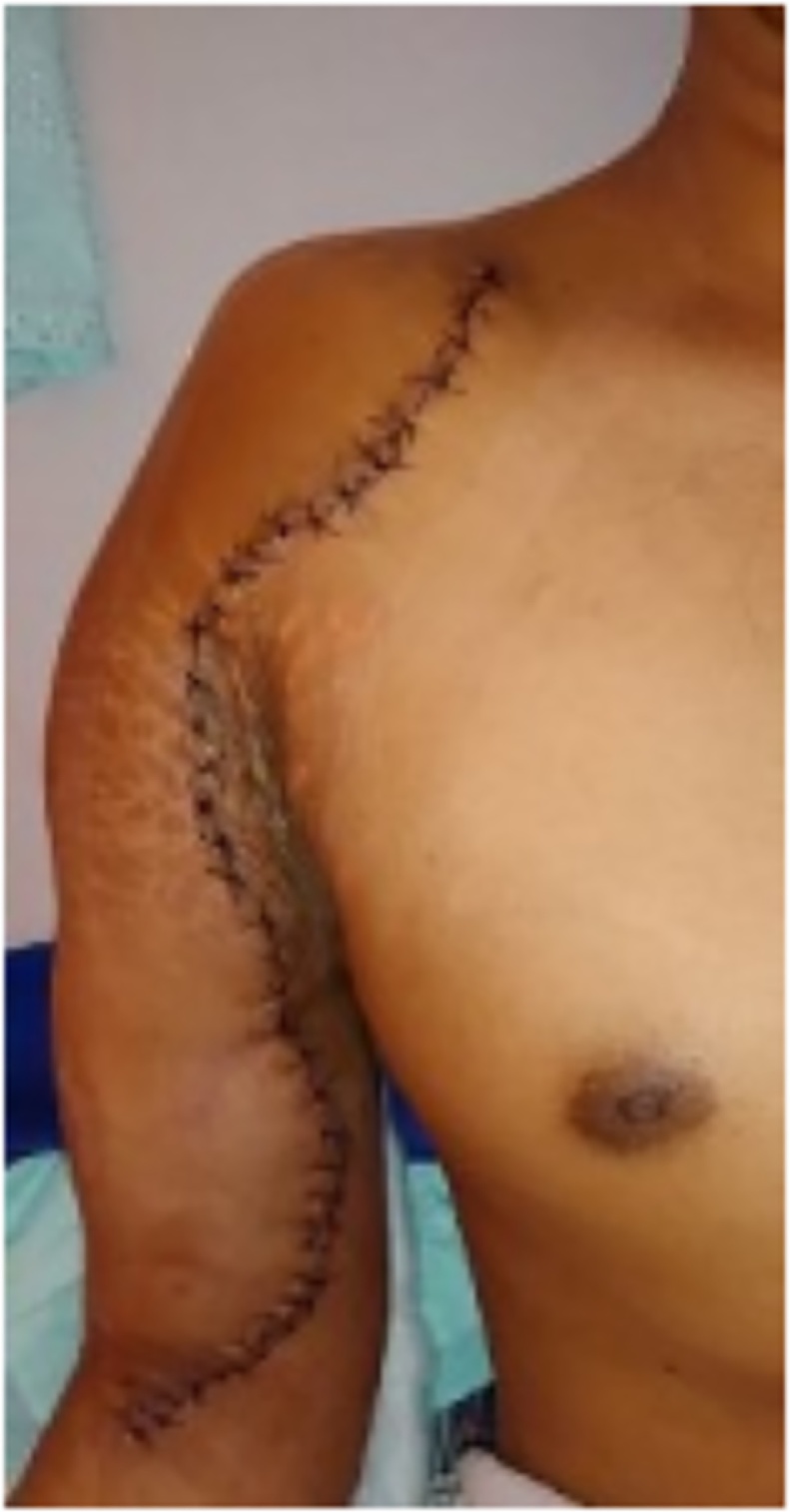
Postoperative clinical view.

The outcome was evaluated 6 months post operatively. The shoulder range of motion was 25° flexion and 15° extensions, and 10° abductions.

## 3 Discussion

GCT of bone typically shows as an epiphyseal, eccentric, expansive lytic lesion with a ‘soap-bubble appearance. MRI is useful to assess extracortical spread and intramedullary extension. The histo morphologic key feature is multinucleated giant cells up to 100 nuclei that have prominent nucleoli. Surrounding mononuclear and small multinucleated cells have nuclei similar to those in the giant cells; this distinguishes GCT of bone from other osteogenic lesions that have benign osteoclast-type giant cells. In this study, MRI provided excellent depiction showing an expansile lytic lesion suggesting the diagnosis of cutaneous GCT Campanacci 3, which was later, confirmed by biopsy. Surgery is the treatment of choice. Curettage is usually combined with cementing or bone grafting. Hemi-articular and total elbow allografts have been used for reconstruction of the defects following tumor excision, but the complication rates are high, and these techniques are reserved as salvage procedures following failed total elbow arthroplasty. Despite being frequently performed in this size and vascularity of tumor, wide resection is still associated with higher rates of surgical complications. Meticulous surgical technique and adequate exposure of the lesion are mandatory to obtain complete removal of tumor. The margin of tumor was determined through plain radiograph and MRI. Limitation of bony involvement and additional 2.5 cm margin are considered to be safe in wide margin resection [22]. The wide resection that we performed showed the margin excision was tumor free. There was a destruction of shoulder joint in this case necessitating wide resection compared to intra lesion curettage. If marginal/ wide excision is chosen as the treatment of the lesion, either primarily or in recurrence, then reconstruction necessarily implies reconstruction of the joint [13]. The presence of extensive bone destruction may be requiring some form of stabilization during surgery. We inserted axial pedicle screw in glenoid, pedicle mono axial in intramedullary distal humerus and rod application. Pedicle screw and rod system has a simple design, an uncomplicated procedure and can shorten the operation time, thus reducing the complication rates [23]. Wide resection and total elbow arthroplasty enables good functional outcome and lower risk for recurrence. Total elbow arthroplasty is a viable option, as it provides good pain relief and functional improvement with lower complication rates.

## 4 Conclusion

In summary, this was a case of a Giant cell tumor of the proximal humerus Campanacci 3. The biopsy and MRI provided excellent depiction in suggesting the diagnosis of GCT. We performed wide excision and reconstruction type 1B with pedicle screw and rod system. The procedure provided excellent local control as the outcome was good both aesthetically and functionally. Compared to endo prosthesis, reconstruction using pedicle screw and rod system provided acceptable outcome with shorter duration of surgery and lower cost. It is most effective especially in developing countries including Indonesia.

## Conflicts of interest

None.

## Funding

This research did not receive any specific grant from funding agencies in the public, commercial, or not-for-profit sectors.

## Ethical approval

Ethical approval has been given by The Ethics Committee of the Faculty of Medicine, University of Indonesia. Number 330/UN2.F1/ETIK/2017.

## Consent

Written informed consent was obtained from the patient for publication of this case report and accompanying images. A copy of the written consent is available for review by the Editor-in-Chief of this journal on request.

## Author contribution

First author: Yogi Prabowo.

Corresponding author: Irsan Abubakar.

Yogi Prabowo contributed to perform the operation, data collection, analysis and interpretation, manuscript drafting, revising, and approval for publishing; Irsan Abubakar contributed to assist the operation, data collection, analysis and interpretation, manuscript drafting, revising, and approval for publishing.

## Registration of research studies

n/a.

## Guarantor

Irsan Abubakar.

## Provenance and peer review

Not commissioned, externally peer-reviewed.
